# Identification of the *CKM* Gene as a Potential Muscle-Specific Safe Harbor Locus in Pig Genome

**DOI:** 10.3390/genes13050921

**Published:** 2022-05-21

**Authors:** Youcai Xiong, Rongzhi Zhuang, Guangxing Zhao, Yanwen Liu, Yinyu Su, Wei Wang, Xiaoning Xi, Yanyu Yang, Xiaosong Han, Shengsong Xie, Heng Wang, Xinyun Li, Bo Zuo, Shuhong Zhao, Zheng Feng, Jinxue Ruan

**Affiliations:** 1 Guangdong Provincial Key Laboratory of Animal Molecular Design and Precise Breeding, Key Laboratory of Animal Molecular Design and Precise Breeding of Guangdong Higher Education Institutes, School of Life Science and Engineering, Foshan University, Foshan 528225, China; 2 Key Laboratory of Agricultural Animal Genetics, Breeding and Reproduction of Ministry of Education & Key Lab of Swine Genetics and Breeding of Ministry of Agriculture and Rural Affairs, Huazhong Agricultural University, Wuhan 430070, China; xyc950602@163.com (Y.X.); zzz1351455398@163.com (R.Z.); gxzhaohzau@163.com (G.Z.); liuyanwen2021666@163.com (Y.L.); suyinyu1024@163.com (Y.S.); wwlucky1005@163.com (W.W.); xiaoningxi@webmail.hzau.edu.cn (X.X.); yyy12111@webmail.hzau.edu.cn (Y.Y.); hanxiaosong2011@163.com (X.H.); ssxie@mail.hzau.edu.cn (S.X.); wangheng@mail.hzau.edu.cn (H.W.); xyli@mail.hzau.edu.cn (X.L.); zuobo@mail.hzau.edu.cn (B.Z.); shzhao@mail.hzau.edu.cn (S.Z.); 3 The Cooperative Innovation Center for Sustainable Pig Production—Swine Breeding and Reproduction Innovation Platform, Huazhong Agricultural University, Wuhan 430070, China

**Keywords:** genetically modified pigs, safe harbor locus, ectopic expression, CRISPR/Cas9, homologous directed repair, *CKM* gene

## Abstract

Genetically modified pigs have shown considerable application potential in the fields of life science research and livestock breeding. Nevertheless, a barrier impedes the production of genetically modified pigs. There are too few safe harbor loci for the insertion of foreign genes into the pig genome. Only a few loci (*pRosa26*, *pH11* and *Pifs501*) have been successfully identified to achieve the ectopic expression of foreign genes and produce gene-edited pigs. Here, we use CRISPR/Cas9-mediated homologous directed repair (HDR) to accurately knock the exogenous gene-of-interest fragments into an endogenous *CKM* gene in the porcine satellite cells. After porcine satellite cells are induced to differentiate, the *CKM* gene promoter simultaneously initiates the expression of the *CKM* gene and the exogenous gene. We infer preliminarily that the *CKM* gene can be identified as a potential muscle-specific safe harbor locus in pigs for the integration of exogenous gene-of-interest fragments.

## 1. Introduction

Site-specific transgene integration is of great significance to the study of gene gain-of-function, especially in the areas of biomedical research and agriculture. Before the discovery of various nucleases that can induce DNA double-strand breaks (DSBs), it was time-consuming and laborious to accurately knock foreign genes into the genome [1]. Methods of integration-exogenous genes are becoming increasingly powerful owing to breakthroughs in the development of various artificial nucleases such as Zinc finger nucleases (ZFNs), transcription activator-like effector nucleases (TALENs), and clustered regularly interspaced short palindromic repeats (CRISPR)/CRISPR associated protein 9 (Cas9) [2,3,4]. Among these systems, CRISPR/Cas9 stands out due to its simplicity and efficiency, and it has become the most versatile tool for numerous genetic manipulations in recent years [5]. When the CRISPR/Cas9 system induces DSBs in the genome, the cell’s own repair mechanism initiates two pathways, namely homologous directed recombination (HDR) and non-homologous end joining (NHEJ) [6]. Precise transgene integration is typically achieved by the HDR pathway in the presence of a repair template. Naturally, the efficiency of the foreign gene integration will be related to the type of cell, the structure of the template, and even the choice of site [7,8,9]. Currently, CRISPR/Cas9-mediated homology-directed repair (HDR) has been widely utilized in various fields, including biomedical research, agricultural genetic modification, etc. [10,11].

With the rapid development of gene editing technology, many genetically modified pigs have been prepared and applied to clinical research and livestock breeding [12,13,14]. However, there is still an obstacle that restricts the production of transgenic pigs—there are too few safe harbor loci for foreign gene knock-in. So far, only three sites—*pRosa26*, *pH11* and *Pifs501*—have been identified as pig-source safe harbor loci for foreign gene insertion and have been successfully used to produce transgenic pigs [15,16,17]. In the above studies, the template plasmid may carry excess components which are to be integrated into the genome. This may interfere with the expression of endogenous genes. The *pGAPDH* and *pACTB* genes have been identified as potential safe harbor loci in pigs, but the hindrance is that no genetically modified pigs have been produced [18,19]. Although these safe harbor loci have been identified and successfully applied, it is essential to identify more potential safe harbor loci.

The marker genes of pig skeletal muscle differentiation are critical to the study of pig muscle differentiation mechanisms and human muscle-related diseases. The *CKM* gene initiates translation at the late stage of muscle differentiation and is highly expressed [20]. We infer that the *CKM* gene may serve as a potential muscle-specific safe harbor locus. The foreign gene is expressed together with the *CKM* gene which is equivalent to conditionally knocking in an exogenous gene that is only expressed after muscle differentiation.

In this study, we seek to identify whether the muscle differentiation marker gene *CKM* can serve as a potential muscle-specific safe harbor locus for the integration of foreign genes. By constructing a donor vector containing the P2A-*EGFP* (P2A refers to the self-cleaving 2A peptide) and an efficient sgRNA expression vector, we successfully used CRISPR/Cas9-mediated HDR to integrate the exogenous P2A-*EGFP* fragment into the pig genome. After pig muscle satellite cells are induced to differentiate, the *EGFP* glows normally. Our results provide an alternative strategy to integrate exogenous genes in pig genome and preliminarily confirm that the *CKM* gene can serve as a potential muscle-specific safe harbor locus.

## 2. Materials and Methods

### 2.1. Plasmids

The CKM-specific single guide RNA (sgRNA) was designed via the website: https://sourceforge.net/projects/crispr-ofnder-v1-2// (accessed on 8 October 2020). Oligonucleotides coding for the sgRNAs were annealed and assembled into a linearized PX330 vector (addgene, #42230) according to the method described by Zhang at the Broad Institute of MIT [5]. The oligonucleotides coding for the sgRNAs were denatured using a thermocycler with the following program: 95 °C, 5 min; 65 °C, 30 min; and holding at 4 °C. Then, the annealed oligos were ligated with BbsI-digested PX330 vector and subsequently, the ligation mixture was transformed into Escherichia coli DH5α competent cells (TakaRa, Otsu, Japan). sgRNA oligodeoxynucleotides are listed in the Supplementary Table S1.

The donor vector (pCDNA3.1-*CKM*-EGFP-KI-donor) was constructed using pCDNA3.1 (+) as the backbone (Figure S1). The homologous arms, EGFP sequence, antibiotic resistance G418 sequence, and P2A sequence were joined together via standard overlapping PCR and then inserted between the Hind III and Kpn1 sites of the pCDNA3.1 (+) vector obtained from Addgene (plasmid #99534). The left and right homologous arms for homologous recombination events at the CKM locus were 800 bp long. Detailed donor vector sequences are listed in the Supplementary Materials.

### 2.2. Quantitative RT-PCR (qPCR)

To identify the expression level of the *CKM* gene in different tissues of pig, total RNAs obtained from the samples were converted into cDNA using the PrimeScriptTM RT reagent Kit with gDNA Eraser (Perfect Real Time) (TaKaRa, Otsu, Japan). The qPCR was performed using the SYBR^®^Green Real-time PCR Master Mix (Toyobo Co., Ltd., Osaka, Japan) on the CFX384 Touch^TM^ Real-Time PCR Detection System following the manufacturer’s instructions (Bio-Rad, Hercules, CA, USA). The Oligo7 Primer Analysis software (Molecular Biology Insights, Inc., Cascade, CO, USA) was applied to design and evaluate the primers for gene validations. The specific primer sequences used for qPCR are shown in Supplementary Table S1 (q-PCR-F/q-PCR-R). The qPCR was performed as follows: 1 cycle at 95 °C for 10 min; 45 cycles at 95 °C for 10 s, 60 °C for 10 s, 72 °C for 15 s; 1 cycle at 72 °C for 2 min and 4 °C for 2 min.

### 2.3. Cell Culture and Transfection

PK-15 and 3D4/21 cell lines were maintained in DMEM supplemented with 10% fetal bovine serum (Hyclone, Logan, UT, USA) and 1% penicillin/streptomycin (Life Technology, Rockville, MD, USA). Cell lines were maintained at 37 °C and 5% CO_2_ in a humidified incubator. At 24 h before transfection, PK-15 and 3D4/21 cell lines were seeded into 6-well plates. When 70–80% confluent, the cells were co-transfected with PX330 plasmid and donor vector (at ratio = 1:1) using the recommended jetPRIME amount. All transfection steps were conducted in accordance with the instructions of the jetPRIME in vitro DNA and siRNA transfection reagent PROTOCOL (PolyPlus, B180306).

### 2.4. T7EN1 Detection Assay and Sequencing

To verify the activity of the sgRNAs, we performed the T7 endonuclease1 (T7EN1) assay. We transfected the constructed sgRNA expression vector into PK15 cells using the recommended jetPRIME amount. After transfection, cells were incubated at 37 °C for 48 h. Then, genomic DNA was extracted using the TIANapm Genomic DNA kit (TIANGEN, Beijing, China). We amplified the targeted region with the PCR programs as follows: 95 °C, for 5 min; 34 cycles of 95 °C for 2 min, 60 °C for 30 s, and 72 °C for 45 s; 72 °C, 2 min; 4 °C, 2 min. The PCR products were purified using the MiniBEST DNA fragment purification kit (TaKaRa, Otsu, Japan) under the manufacturer’s instructions. To induce the mismatches between heteroduplexes of the WT and mutant alleles, we melted and reannealed the purified PCR products with a temperature program: 95 °C for 10 min, 95 °C to 85 °C ramping at −2 °C/s, 85 °C to 25 °C at −0.25 °C/s, and 15 °C hold for 2 min. The reannealed products were digested by T7EN1 (NEB) enzyme at 37 °C for 15 min. The digested products were analyzed on 2% agarose gels stained with Gel-Rad, and then quantified by densitometry using ImageLab software (Bio-Rad, Hercules, CA, USA). The online website tool TIDE was used to calculate the indel rate [21].

### 2.5. Screening of Monoclonal Cells

To knock the GFP gene into the pig genome, we co-transfected the PX330 plasmid and the targeting donor plasmid (at radio = 1:1) into the cells. To detect the efficiency of knock-in, we used the limiting dilution analysis to screen monoclonal cells. We aspirated the cell line that needed to be cloned from the culture well, added a small amount of medium to dilute, and pipetted the right amount of cells to a large petri dish when there were only 1–2 cells in the view, all of which were transferred to a 96-well plate petri dish. After about 10 days, we extracted the genomic DNA and identified the genotype per clone.

### 2.6. Off-Target Analysis of sgRNA

We used the website (http://crispr.mit.edu/ (accessed on 17 March 2021)) to predict potential off-target sites (OTS) of the CKM-sgRNA and selected the top 7 sites from the genomic DNA. After PCR amplification of each potential OTS, Sanger sequencing was performed to determine whether any point mutation occurred. The primers were listed in Table S1.

## 3. Results

### 3.1. Identification of the Expression of the CKM Gene in Various Tissues

**Figure 1:**
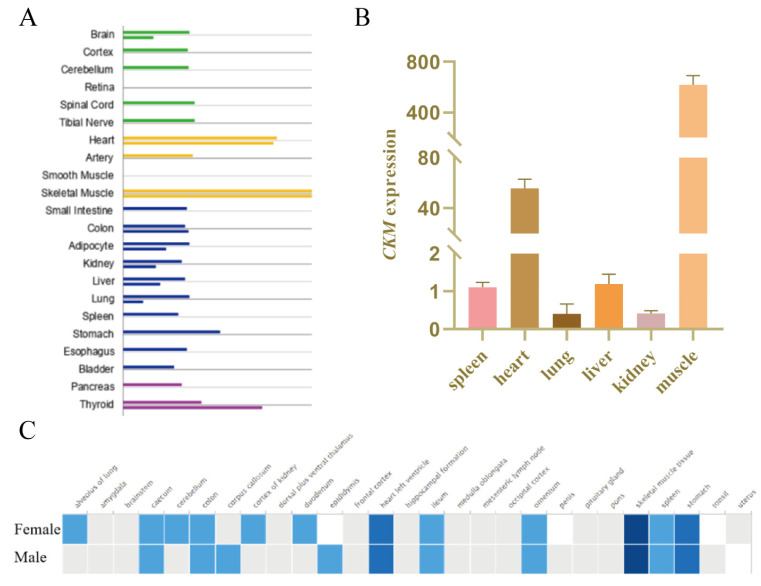
Expression of the *CKM* gene in different tissues of human and pig. (**A**) Gene Cards database analysis shows that the *CKM* gene expression level is higher in skeletal muscle and myocardium. (**B**) Quantitative Real-time PCR to detect the expression of the *CKM* gene in pig heart, liver, spleen, lung, kidney and muscle tissues. (**C**) Expression Atlas database analyzes the expression of the *CKM* gene in boars and sows.

The *CKM* gene is specifically expressed in muscle tissue and has a higher expression level in skeletal muscle and myocardium. Analyzing the expression of the *CKM* gene in different human tissues and cells through the Gene Cards database (https://www.genecards.org/ (accessed on 13 October 2020)), we found that the *CKM* gene is widely expressed in different human tissues and cells, and it is highly expressed in the skeletal muscle, thyroid gland and cardiac muscle (Figure 1A). After analyzing the expression of the *CKM* gene in different tissues of pigs in the Expression Atlas database (https://www.ebi.ac.uk/gxa/home (accessed on 13 October 2020)), we discovered that the *CKM* gene has a higher expression level in the skeletal muscle tissues of boars and sows (Figure 1C). Furthermore, we used Quantitative Real-time PCR to detect the expression of the *CKM* gene in the heart, liver, spleen, lung, kidney, muscle and other tissues of pig. The expression of the *CKM* gene in skeletal muscle and myocardium is significantly higher than that in other tissues (Figure 1B).

### 3.2. Establishment of the Knock-In Reporter System in Pig Genome

The purposes of this study are to establish a reporter system to knock the exogenous EGFP gene into the downstream region of the *CKM* gene and identify whether the *CKM* gene provides a novel alternative safe harbor locus in the pig genome.

**Figure 2:**
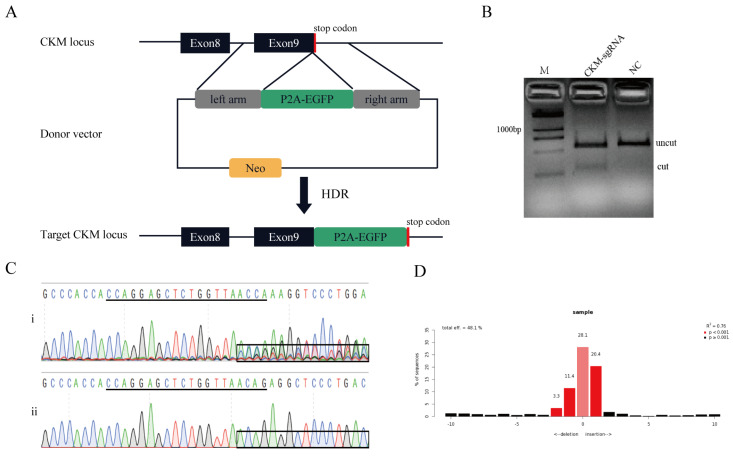
Schematic diagram of CKM locus gene targeting. (**A**) Exons of CKM are shown as black boxes, and the stop codon is shown as a red box. The black triangle box between exon 9 and the stop codon represent the sgRNA target site. The targeting donor was created according to the cleavage location of Cas9 and carried 800 bp regions of homology to the CKM sequence astride the cleavage site. The yellow box represents the resistance gene. (**B**) Identification of the activity of CKM-sgRNA by T7EN1 cleavage assay. NC, Negative Control; M, Marker, DL2000. (**C**) Sequence analysis showing that the presence of multiple peaks after the targeted site in the sequencing curves clearly distinguishes. (**i**) mutants, (**ii**) wild type. SgRNA sequence is underlined in black. (**D**) TIDE analysis of indel rate of the CKM-sgRNA.

Thus, we have developed a reporter system targeting the *CKM* locus in the pig genome. The donor vector was generated to carry a promoter-less 2A-EGFP sequence flanked by two regions of homology. When HDR-mediated knock-in events occurred, the P2A-EGFP fragment was inserted in frame with the endogenous *CKM* coding sequence, and because the self-cleaving P2A peptide exists, the CKM and EGFP can be expressed separately (Figure 2A).

One sgRNA was designed to target the upstream of the stop codon of the *CKM* gene. The sgRNA expression vector involves two parts: the sgRNA oligodeoxynucleotides (Table S1) and the PX330 vector backbone. The plasmid sequence was verified after transformation into E. coli. The activity of sgRNA was confirmed prior to co-transfection. We transfected the PX330-CKM-sgRNA plasmid into PK15 cells, extracted the genomic DNA of these cells, and then amplified the target region. Amplification primer information (F1/R1) is supplemented in Table S1. The PCR products were used to identify the activity of sgRNA via T7EN1 cleavage assay, and the results of T7EN1 cleavage and DNA sequencing prove that the sgRNA has high activity (Figure 2B,C). The frequency of the indels was calculated by the online software TIDE, and results showed that the ratio of the indels was 48.1% (Figure 2D).

Potential off-target sites (OTS) were predicted by a website: http://crispr.mit.edu/ (accessed on 17 March 2020). In total, 7 potential OTS with less than four mismatches to the CKM-sgRNA were selected (Table 1). Sanger sequencing was performed and the results indicated that no mutation occurred in the potential off-target loci. The results also demonstrated that there was no disruption to the sequence of all the OTS. The specific sequencing information of each potential off-target site is displayed in the Supplementary Materials (Figure S2).

### 3.3. Assay of the Knock-In Efficiency in Pig Genome

**Figure 3:**
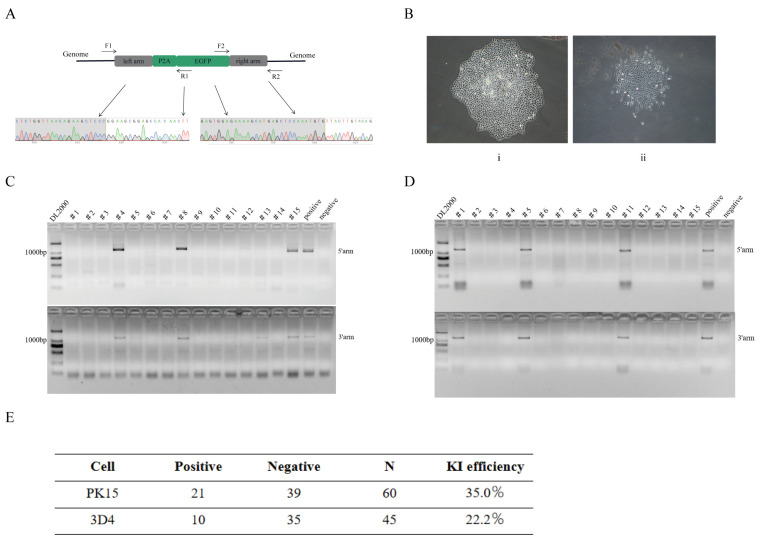
Detection of knock-in efficiency in pk-15 and 3D4/21 cells. (**A**) Nucleotide sequence analysis of junctions between endogenous and exogenous DNA corresponding to HDR events. F1/R1 and F2/R2 primers were used to amplify specific regions of the 5′arm and 3′arm. (**B**) pk-15 monoclonal cell (**i**) and 3D4/21 monoclonal cell (**ii**). (**C**) PCR amplification of homologous arm sequences of 15 pk-15 monoclonal cells. (**D**) PCR amplification of homologous arm sequences of 15 3D4/21 monoclonal cells. (**E**) knock-in efficiency statistics.

To calculate the knock-in efficiency, we co-transfected two plasmid vectors (the donor vector and the sgRNA expression vector) in PK-15 and 3D4/21 cells and then enriched them with 1 mg/mL G418. After 4 days, all wild-type cells died. We collected the surviving cells of the transfection group and picked out monoclonal cells. After culturing them for 10 days, an independent cell cluster was observed (Figure 3B). We collected some monoclonal cells to extract the genomic DNA and designed two pairs of primers that span the homology arms to amplify the junctions. The detailed primer sequences (F2/R2 and F3/R3) are listed below (Table S1). A total of 60 PK-15-KI monoclonal cells were collected, 21 of which were positive clones. Moreover, we collected 45 3D4/21-KI monoclonal cells, 10 of which were positive clones. The knock-in efficiency reached 35% and 22.2%, respectively (Figure 3C–E). The amplification results of all monoclonal cells are listed below (Figure S3).

To identify whether EGFP was knocked into our desired target area, we sent the amplified products of the homology arms of the positive cells for Sanger sequencing. The results showed that the exogenous EGFP gene was precisely knocked into the downstream region of the *CKM* gene (Figure 3A).

### 3.4. Identification of the CKM Gene Knock-In System in Muscle Satellite Cells

**Figure 4:**
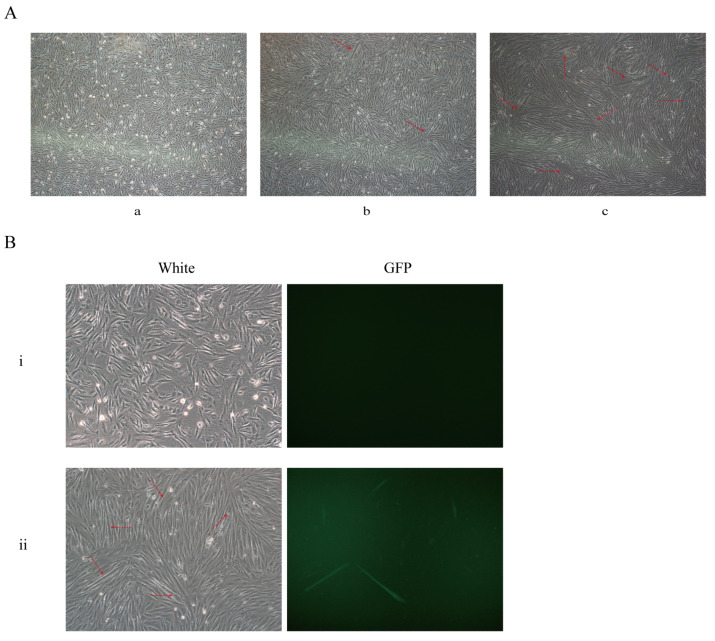
The exogenous EGFP gene can be expressed under the drive of the endogenous CKM promoter. (**A**) The differentiation of porcine muscle satellite cells. (**a**) Proliferative porcine skeletal muscle satellite cells, (**b**) continuously induced differentiation for 24 h, (**c**) continuously induced differentiation for 48 h. Red arrow marks where myotubes are formed. (**B**) Co-transfection of porcine skeletal muscle satellite cells. (**i**) No EGFP expression was observed 48 h after co-transfection, (**ii**) EGFP expression was observed after 48 h of continuous induction and differentiation.

The strategy of this study is to insert the EGFP sequence downstream of the *CKM* locus. I EGFP gene can be expressed in frame with the *CKM* gene. We co-transfected porcine satellite cells with two plasmid vectors and did not observe EGFP expression after 48 h (Figure 4B). It is reported that the *CKM* gene is activated and expressed in skeletal muscle at the end of muscle differentiation [20,22]. We infer that this is why the *GFP* gene is not expressed.

In the next step, we used a differentiation medium to induce differentiation. After 48 h of differentiation, the cells began to fuse into myotubes (Figure 4A). Meanwhile, we observed the expression of EGFP protein (Figure 4B). The above results indicate that when the *CKM* gene is expressed, EGFP can be expressed normally under the drive of the *CKM* gene promoter.

## 4. Discussion

Recently, CRISPR/Cas9 technology has been widely used in various fields, such as clinical research, species genetics, and agriculture [23,24,25,26]. A variety of targeted integration methods mediated by the CRISPR/Cas9 system have been reported [27,28]. Although there are many methods for the site-specific integration of foreign genes, these methods are based on the two repair mechanisms of HDR and NHEJ. Some technologies based on NHEJ have significantly improved integration efficiency, but also introduce undesired insertions and deletions (indels), such as HMEJ and HITI [29,30,31]. Other methods based on HDR are slightly more efficient than the original CRISPR/Cas9-mediated HDR, but they are more time-consuming and laborious, including the modification of the template structure and the Cas9 protein [8,32]. Taking all the factors into consideration, we prefer the original CRISPR/Cas9-mediated HDR to integrate foreign genes into specific sites. In this study, the efficiency of knock-in in PK15 and 3D4/21 cells was evaluated The results for both cell lines were greater than 20% which is quite considerable. 

It was not until 2014 that the pig’s first safe harbor locus *pRosa26* was identified [15]. Subsequently, Ruan et al., proved *pH11* as a safe harbor locus which can be used for foreign gene insertion [16]. In 2018, Ma et al., identified *Pifs501* as another available safe harbor locus [17]. Compared with *pRosa26* locus, *Pifs501* has an equal effect. For the above-mentioned safe harbor loci, it is inevitable to introduce redundant element components to the template plasmid, including the promoter and polyA sequences. To some extent, these elements are excessive and may interfere with the expression of endogenous genes. Our laboratory has identified two housekeeping genes, *GAPDH* and *ACTB*, as potential alternative safe harbor loci that can be applied. We have verified them in three pig cell lines [18,19], although genetically modified pigs were not produced. Following our strategy, the targeting vector does not carry any excess component, and the exogenous fragments are integrated downstream of the stop codon of the potential safe harbor locus without disrupting the expression of the endogenous gene. Therefore, we have similarly identified that the *CKM* gene can be used as a potential safe harbor locus. it is only expressed in the late stage of muscle differentiation. Pig-source safe harbor loci cannot easily meet the growing demand for genetically modified pigs. It is critical to identify more safe harbor loci that can be used for ectopic expression of foreign genes.

The off-target effect of the CRISPR system has become a concerning problem in many fields, especially for the production of genetically modified animals [33,34]. In this study, the sgRNA-dependent off-target effect has been considered and identified. According to the results of sequencing, no off-target effect has been detected among the 7 potential off-target sites, indicating the safety of this sgRNA for producing genetically modified animals.

The *CKM* gene is only driven by its promoter at the final stage of muscle differentiation, so its expression has temporal and spatial specificity. If the gene-of-interest fragment is integrated downstream of the stop codon at this site, the exogenous fragment will also have specific temporal and spatial expression. There is no doubt that this is of great significance to the study of certain gene functions, and it is also a new strategy for studying muscle-related gene functions.

In conclusion, our results provide preliminary verification that the *CKM* gene may be served as a potential muscle-specific safe harbor locus in the pig genome. Driven by the endogenous promoter of the *CKM* gene, the exogenous fragment can be expressed simultaneously with the *CKM* gene. Our research also provides a novel strategy for knocking transgene into the pig genome, which is conductive to studying the function of a specific gene. Similar strategies and methods may also be applied to identify other loci.

## Figures and Tables

**Table 1:** Analysis of potential off-target sites. Seven potential off-target sites were selected, and the off-target results were identified by PCR- sequencing. Blue letters indicate the PAM (protospacer adjacent motif) sequence. Red letters mark differences in sgRNA compared with the target sequence. Indel column shows the detected off-target results.

#	Predicted OTS	Sequence (5′ to 3′)	Indel
	CKM-sgR1	CCAGGAGCTCTGGTTAACAG AGG	
1	Predicted-OFF-Target1	CCTGGAGCTCCGGTTAGCAG GGG	NO
2	Predicted-OFF-Target2	CGAGGAGGTCTGGCTAACAG GGG	NO
3	Predicted-OFF-Target3	GCAGGAGCTCTGGATGACAG TGG	NO
4	Predicted-OFF-Target4	GCAGGAGCTCTGTTTATCAG TGG	NO
5	Predicted-OFF-Target5	CCTGGAGCTCTGGTTGGCAG TGG	NO
6	Predicted-OFF-Target6	CCAGGAGCTCTGGGGCACAG AGG	NO
7	Predicted-OFF-Target7	CCAGGAGCTCTGGGTGGCAG TGG	NO

## Supplementary Materials

The following supporting information can be downloaded at: https://www.mdpi.com/article/10.3390/genes13050921/s1, Figure S1: Vector map of targeting donor (pCDNA3.1-*CKM*-EGFP-KI-donor); Figure S2: Sequencing information of each potential off-target site; Figure S3: Amplification results of all monoclonal cells; Table S1: All primers used in this study.
