# Supplementary material for: Identification of the CKM Gene as a Potential Muscle-Specific Safe Harbor Locus in Pig Genome
Source: Genes (Basel). 2022 May 21;13(5):921. doi: 10.3390/genes13050921 (PMC9140944; doi:10.3390/genes13050921)
Supplement: Supplementary file 1 [file genes-13-00921-s001.zip › genes-1675610-supplementary.pdf]

Figure S1

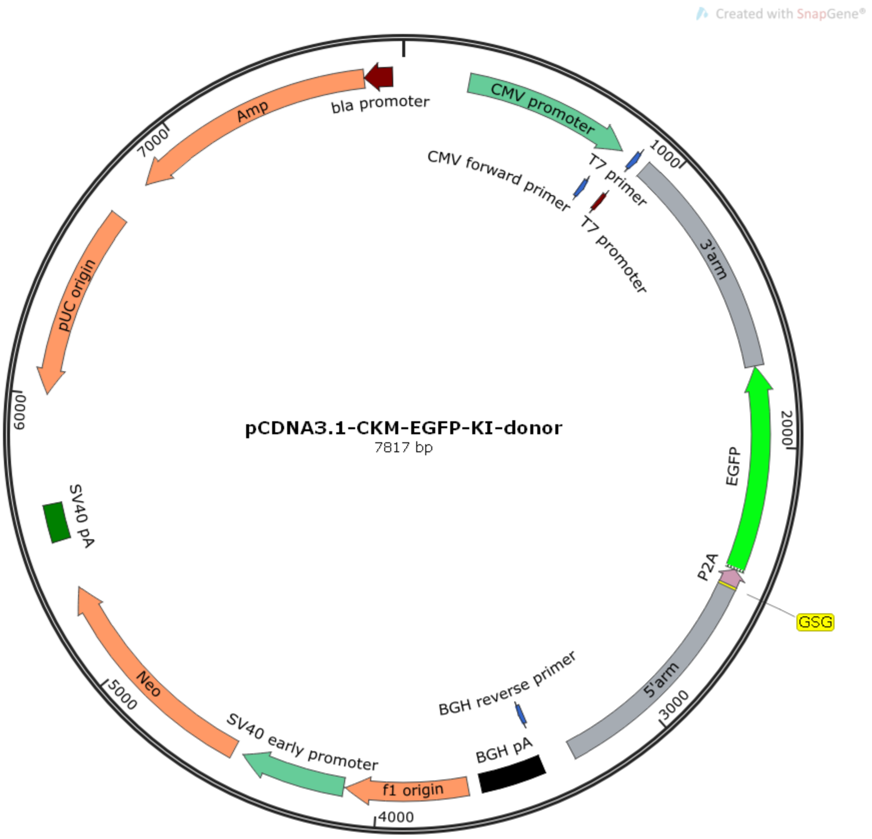

Figure S1. Vector map of targeting donor (pCDNA3.1-*CKM*-*GFP*-KI-donor).

Detailed sequence of the donor vector(the sequences of homolog arms and P2A-*EGFP* fragments are marked in different colors corresponding to the vector map mentioned above):

gacggatcgggagatctcccgatcccctatggtgcactctcagtacaatctgctctgatgccgcatagttaagcca
gtatctgctccctgcttgtgtgttggaggtcgctgagtagtgcgcgagcaaaatttaagctacaacaaggcaagg
cttgaccgacaattgcatgaagaatctgcttagggttaggcgttttgcgctgcttcgcgatgtacgggccagatat
acgcgttgacattgattattgactagttattaatagtaatcaattacggggtcattagttcatagcccatatatggag
ttccgcgttacataacttacggtaaatggcccgcctggctgaccgcccaacgacccccgcccattgacgtcaataa
tgacgtatgttcccatagtaacgccaatagggactttccattgacgtcaatgggtggagtatttacggtaaactgc
ccacttggcagtacatcaagtgtatcatatgccaagtacgccccctattgacgtcaatgacggtaaatggcccgcc
tggcattatgcccagtacatgaccttatgggactttcctacttggcagtacatctacgtattagtcatcgctattacc
atggtgatgcggttttggcagtacatcaatgggcgtggatagcggtttgactcacggggatttccaagtctccacc
ccattgacgtcaatgggagtttgttttggcaccaaaatcaacgggactttccaaaatgtcgtaacaactccgcccc
attgacgcaaatgggcggtaggcgtgtacggtgggaggtctatataagcagagctctctggctaactagagaac
ccactgcttactggcttatcgaaattaatacgactcactatagggagacccaagctggctagcgtttaaacttaag
cttACCACAGCCACAGCAACCGGATCCTTAACCCACTGAGCAAGGCCAGGGAT
TGAACCCGCAACCTCATGGTTCCTAGTCAGATTCGTTAACCACTGAGCCATG
ACGGGAACTCCCAGATATTTCTTTAAGGTGGGAATTTCAGGGTGTAAGGAG
CAAAAGAGTATACAGAGAGATCATGAGAAAAGAACTACAAAGATGAGCCTT

CAGCAAATGGTATGCATTTGTTTGAAGACTTGCATATAAAGGAGAGTATTCT
AGGAGACGTGGGGAGCCATGGTAGGTTCTTGAGCAAAGTAAGTGAAGTTTA
TGGGGGAAAAACAGGTAGCACCTGCAGAGGGCTGAAGTCTGGAGGCTAAT
GAAAAGGTTAATGAGATAGATCATGATGAGCTGTATGGGAGATCTCAACCT
GGGAGCCAAGTTCAAGTCTCAGACATTTCATTTGGCCTGTTTCTTCCTCATG
TGGACCCTGTGTTTGAGATCTAACCTGAATGACTGGCAGCTGAGGTTGGGA
AGGACCCAAGCTTGGTTGTGGGACTCTGGCCACACCTGAGTCTCGGATTCC
ATGCAGAGCCTTGTAATGTTTGAATTAGACTCTGTAGGGTAACTGTGCCTCA
TTCTAGAGAAGGGATAAATATGCCTAGCCCTAGGACATGGCTAGAGAGGCA
TGGCGGCTTCGAGGTCACACCGTAGCCAATTGTGAAAGTCCCTCCTCCTCCC
CCAATAACTATGGCATCAATGCTAAGGCCACCATTGCTTTATTTGTTGCTTT
GAGTGGAGAAAAGCATGAGCTCCAAATGTGTTACTTGTACAGCTCGTCCAT
GCCGAGAGTGATCCCGGCGGCGGTCACGAACTCCAGCAGGACCATGTGATC
GCGCTTCTCGTTGGGGTCTTTGCTCAGGGCGGACTGGGTGCTCAGGTAGTG
GTTGTCGGGCAGCAGCACGGGGCCGTCGCCGATGGGGGTGTTCTGCTGGTA
GTGGTCGGCGAGCTGCACGCTGCCGTCCTCGATGTTGTGGCGGATCTTGAA
GTTCACCTTGATGCCGTTCTTCTGCTTGTCGGCCATGATATAGACGTTGTGG
CTGTTGTAGTTGTACTCCAGCTTGTGCCCCAGGATGTTGCCGTCCTCCTTGA
AGTCGATGCCCTTCAGCTCGATGCGGTTCACCAGGGTGTCGCCCTCGAACTT
CACCTCGGCGCGGGTCTTGTAGTTGCCGTCGTCCTTGAAGAAGATGGTGCG
CTCCTGGACGTAGCCTTCGGGCATGGCGGACTTGAAGAAGTCGTGCTGCTT
CATGTGGTCGGGGTAGCGGCTGAAGCACTGCACGCCGTAGGTCAGGGTGGT
CACGAGGGTGGGCCAGGGCACGGGCAGCTTGCCGGTGGTGCAGATGAACT
TCAGGGTCAGCTTGCCGTAGGTGGCATCGCCCTCGCCCTCGCCGGACACGC
TGAACTTGTGGCCGTTTACGTCGCCGTCCAGCTCGACCAGGATGGGCACCA
CCCCGGTGAACAGCTCCTCGCCCTTGCTCACCATGGGGCCGGGGTTCTCCTC
CACGTCGCCGGCCTGCTTCAGCAGGCTGAAGTTGGTGGCTCCGCTTCCGGG
AGCTTCTGTTAACCAGAGCTCCTGGTGGTGGGCGGAGCTCAGGGATAATGA
AAGGTGAATGTGAAGGGAGGGTTTTCATTGGCCAGAACCCAGAGAATGGAG
CCCATTGGCTGGAACTCTGGTTGGAACTGGAATTCGATGAAGAGTGGGGAG
AGAGCCCCGGGTTGACTGACCCTTGTCAGCCAGATTCTGGAAGGAAGGGGC
GGGCCTTCGATGGGCTTGGTCCTCCCTTTGCCCTGGGCTCCAGCAGTCGGCT
GGGAGCCTACTTCTGGGCGGGGATCATGTCGTCGATGGACTGGCCTTTTTC
CAGCTTCTTCTCCATCTCCACCATGAGCTTCACACCATCCACCACCAGCTGCA
CCTGTTCTACCTCCGACGAGCCCAGCCGATCGGCGTTGGATACATCGAACAC
TGAGCCCACAGCGGCTGTGTCCACGCCACCTGCGGGAGGAGGAGGCCGCGG
CATGAGAAGGGAGCAGGGATGCAGGAGCGGCCCCCGTGCCAAGTGCGACA
GCCACACGTACTGAGTGAGTGCCTACTGTGTATGGGTTAAGTGCACGCAGA
GTTTCCTCAGCATCTGTCTTTTCACGGGCCCACATCTCTGGCTGTAGGTACC
CCACCCTTCAACCATCCAACAAGAAAATACAGCGTGATGGGTAGGAGAGTC
GAGCTTTGGAGCCGAGCTGCCTGGGGTTGAATCCAGACCCTCCTCCCCTTTA
CAAATAGGAAAATAAGGCCCCGAGGCGGCCAGGGACATACTCGGTGTGTAT
TACACAGCCATGATGATGTACTCATTCggtaccgagctcggatccactagtccagtgtggtgg
aattctgcagatatccagcacagtggcggccgctcgagtctagagggcccgtttaaacccgctgatcagcctcga

ctgtgccttctagttgccagccatctgttgtttgcccctcccccgtgccttccttgaccctggaaggtgccactccca
ctgtcctttcctaataaaatgaggaaattgcatcgcattgtctgagtaggtgtcattctattctggggggtggggtg
gggcaggacagcaagggggaggattgggaagacaatagcaggcatgctggggatgcggtgggctctatggctt
ctgaggcggaaagaaccagctggggctctagggggtatccccacgcgccctgtagcggcgcattaagcgcggc
gggtgtggtggttacgcgcagcgtgaccgctacacttgccagcgccctagcgcccgctcctttcgctttcttccctt
cctttctcgccacgttcgccggctttccccgtcaagctctaaatcgggggctccctttagggttccgatttagtgcttt
acggcacctcgaccccaaaaaacttgattagggtgatggttcacgtagtgggccatcgccctgatagacggtttt
cgccctttgacgttggagtccacgttctttaatagtggactcttgttccaaactggaacaacactcaaccctatctcg
gtctattcttttgatttataagggattttgccgatttcggcctattggttaaaaaatgagctgatttaacaaaaattta
acgcgaattaattctgtggaatgtgtgtcagttagggtgtggaaagtccccaggctccccagcaggcagaagtat
gcaaagcatgcatctcaattagtcagcaaccaggtgtggaaagtccccaggctccccagcaggcagaagtatgc
aaagcatgcatctcaattagtcagcaaccatagtcccgcccctaactccgcccatcccgcccctaactccgcccag
ttccgcccattctccgcccc

Figure S2

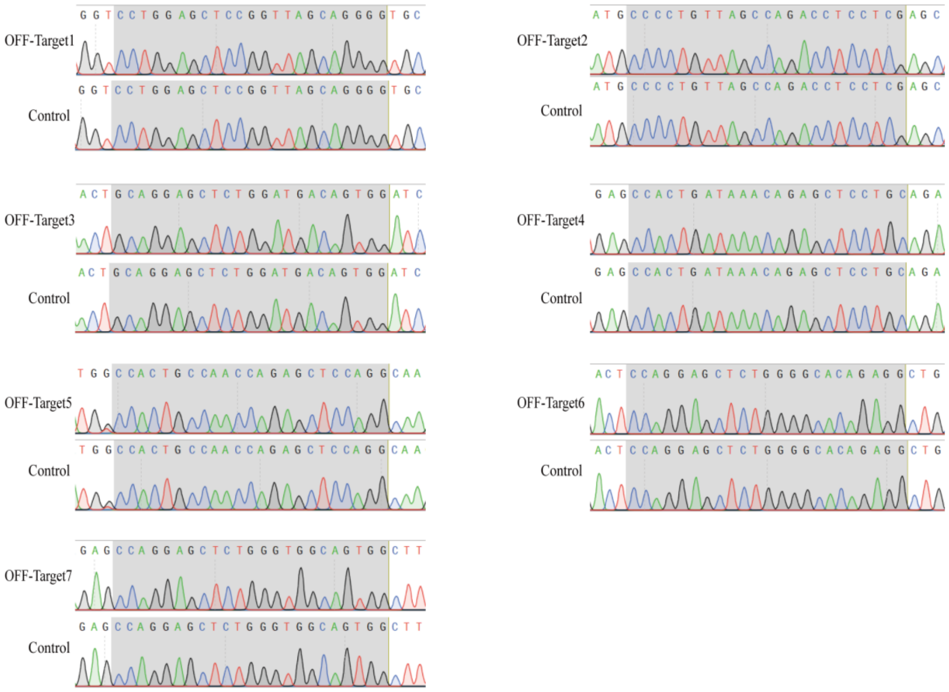

Figure S2. Sequencing information of 7 potential OFF-Target sites.

Figure S3

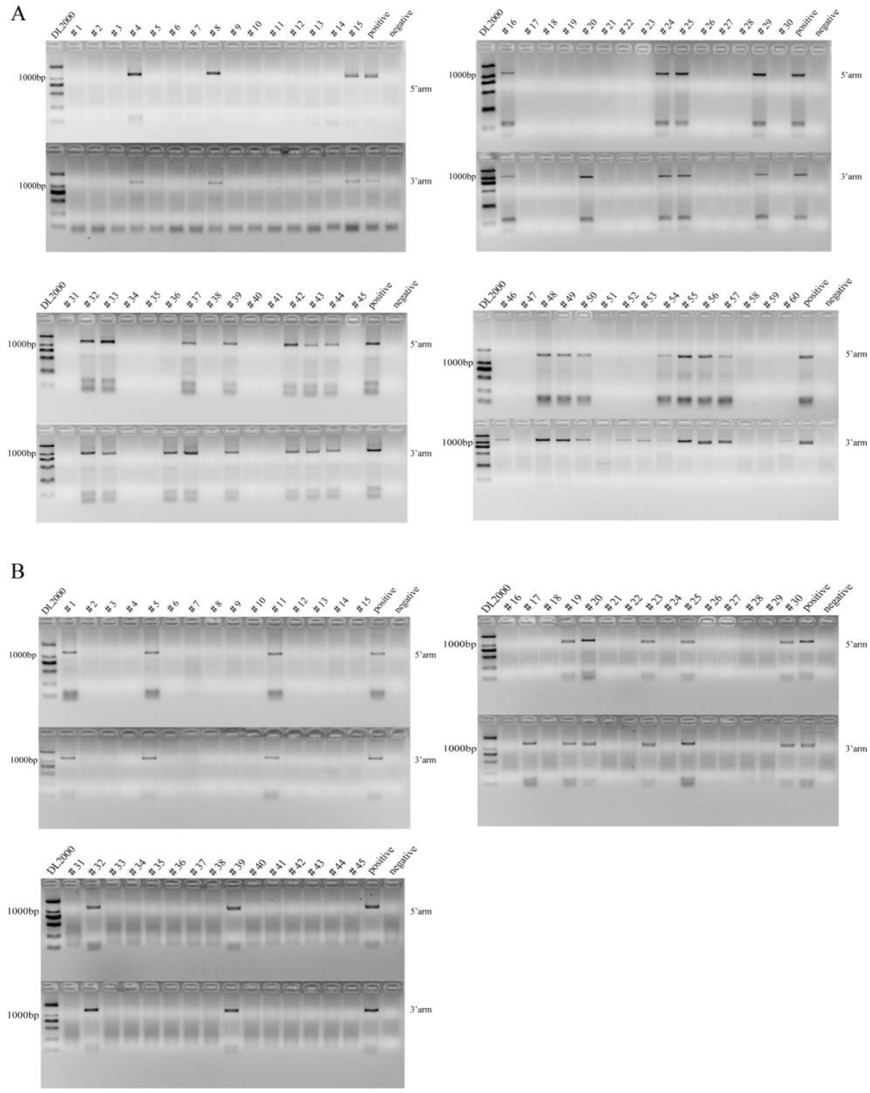

Figure S3. PCR amplification of homologous arm sequences of 60 pk-15 monoclonal cells(A) and 45 3D4/21 monoclonal cells(B).

Table S1. All primers used in this study.

Primer	Sequence (5'-3')
CKM-sgR-F	caccCCAGGAGCTCTGGTTAACAG
CKM-sgR-R	aaacCTGTTAACCAGAGCTCCTGG
CKM-offtarget1-F	ACAGCGAAGTCCACAAAGGT
CKM-offtarget1-R	ACCCACCATCCCTAAAAAGGT
CKM-offtarget2-F	CTGAGCCCCTGAGAGGTACT
CKM-offtarget2-R	AACTAACAGAGGCACGGTGG
CKM-offtarget3-F	AGACAGGGTGTACCTTTCCAT
CKM-offtarget3-R	GCACTGTAAATGGCAGAGCG
CKM-offtarget4-F	TGAAACCCAGCAAACCTGGA
CKM-offtarget4-R	GAGTTGACAGCACTGACCCA
CKM-offtarget5-F	GCCGACTTTAGGCTGTGTCT
CKM-offtarget5-R	GTGTCCAGCAAGACCACTGA
CKM-offtarget6-F	CGAGCTGCACCATTCACAAC
CKM-offtarget6-R	ACATCAGAAGAGGCGCTCAC
CKM-offtarget7-F	GTACCAGATGGCGGTACCTT
CKM-offtarget7-R	CAGGGAGGAAGACAGACCAC
q-PCR-F	CCTCAGCAAGCACAACAACC
q-PCR-R	CCGTGTCGGTCTTGGATGAT
F1	TGATCCCCGCCCAGAAGTA
R1	GAGTCTCGGATTCCATGCAGA
F2	GCATCCCAAGTTCGAGGAGA
R2	GCTGAACTTGTGGCCGTTTAC
F3	AGATCCGCCACAACATCGAG
R3	ATGATTGACGTGGCCAACAC
